# Ranking Research Methodology by Risk — a cross-sectional study to determine the opinion of research ethics committee members

**DOI:** 10.1186/s13643-023-02295-1

**Published:** 2023-09-01

**Authors:** Simon E. Kolstoe, Jennifer Durning, Jennifer Yost, Silviya Aleksandrova-Yankulovska

**Affiliations:** 1 School of Health and Care Professions, University of Portsmouth, Portsmouth, UK; 2 Fitzpatrick College of Nursing, Villanova University, Driscoll Hall 330, Villanova, PA 19085 USA; 3 Massachusetts General Hospital Institute of Health Professions, 36 1st Ave, Charlestown, MA 02129 USA; 4 Faculty of Public Health, Medical University of Pleven 1, Kliment Ochridski str, Pleven, 5800 Bulgaria

**Keywords:** Ethics committees, Institutional review boards, Research methodology, Risk, Harm, Systematic reviews, REC, IRB

## Abstract

**Background:**

When reviewing a protocol, research ethics committees (RECs, equivalent to institutional review boards — IRBs) have the responsibility to consider whether the proposed research is justified. If research is not justified, it can waste participants’ time, researchers’ time and resources. As RECs are not constituted to cover all areas of scientific or academic expertise, it can be difficult for RECs to decide whether research is scientifically or methodologically justified especially in the absence of authoritative (often in the form of systematic) reviews. Where such reviews are absent, some have argued that RECs should insist on a new review of existing evidence as a condition of the REC favourable opinion. However, as RECs review a wide range of research, such requests must be proportionate to the type, and extent, of proposed projects. Risk is one factor that may influence the extent of evidence need for a REC to determine that the new project is justified, but not the only factor. The aim of the work described here was to determine whether REC members and researchers specifically link risk to the type of research methodology, and if so, whether this link could be used to help guide the need for systematic, or other, types of reviews.

**Method:**

We conducted a cross-sectional study, gathering data between November 2020 and January 2021, to examine whether proposed research methodologies impact how RECs perceive risk to participants. We presented 31 research methodologies to REC members and researchers in the form of an international survey.

**Results:**

We collected 283 responses that included both qualitative and quantitative data as to how research methodology impacts perceptions of risk to participants. We used the data to conclude that RECs did see a link between risk and type of research. We therefore constructed a hierarchy of risk with Phase 1 and 2 clinical trials, and clinical psychology/psychiatry intervention studies, at the top (i.e. viewed as most risky).

**Conclusions:**

We discuss whether this hierarchy is useful for guiding RECs as to the level of scientific justification that they should seek when reviewing proposed research protocols, and present a one-page guidance sheet to help RECs during their reviews.

**Supplementary Information:**

The online version contains supplementary material available at 10.1186/s13643-023-02295-1.

## Background

Ethics review conducted by research ethics committees (RECs, or institutional review boards — IRBs in the US) seldom require researchers to produce a systematic summary, or review, of the literature relating to the research question and proposed protocol [1, 2]. Instead, applicants to RECs normally provide a short summary as to the relevance, and thus justification, of their proposed study [3]. However, RECs are historically criticised for not having sufficient specialist expertise to review the methodology of all the different types of projects that are presented to them [4]. They are also often criticised for slow review times and being overly bureaucratic [5]. As a consequence, if they acknowledge their lack of expertise in relation to a certain piece of research, and thus their need for more information, they face the difficult decision of either accepting the research teams justification at face value, which could lead to unethical research, or slow the review process by seeking reassurance by requesting additional reviews including, on occasion, full systematic reviews. Although some argue that it is not really the RECs role to consider research justification or methodology [6], in practice, the ethical aspects of projects can seldom be cleanly separated from the technical details of the research [7]. Bad science is bad ethics.

A formal systematic review can be used as evidence for an objective, up to date, summary of prior findings that researchers can use to justify their research plans. As systematic reviews play an important role in the setting of research priorities, RECs may well expect to see such reviews especially for large and well-funded clinical studies. However, a proportionate approach is needed for other types of studies because, assuming that a systematic review does not already exist, there is often not the time or funding to produce extensive, time-consuming, and expensive, reviews for every research question or protocol. But there is as yet little understanding as to what type of review is needed to justify different types of research.

Between 2018 and 2023, the European Commission funded the evidence-based research (EVBRES) consortium “To encourage researchers and other stakeholders to use an Evidence-Based Research (EBR) approach while carrying out and supporting clinical research – thus avoiding redundant research” [8]. One working group within this project focussed specifically on the role of RECs in encouraging better use of evidence. Following a scoping review (Kolstoe & Munro 2018, student project, unpublished) and 2-year consultation among the EVBRES participants (which included a number of experienced ethics committee members and chairs), the role of risk was hypothesised as an important aspect that REC members take into account when considering the suitability of a researcher’s justification for their proposed project.

The aim of this study was therefore to explore empirically how REC members understand risk in relation to research methodology, by conducting a mixed-methods questionnaire among ethics committee members and others in the research community. We tested whether a feasible hierarchy of research methodologies could be created based upon risk by examining the qualitative data (also collected in the questionnaire) as to how REC members and researchers link the idea of risk to the need for different levels of justification when research is presented to them for review. As the major output of the EVBRES working group, we propose a one-page information sheet that can be used as a guide for RECs when reviewing studies.

## Methods

### Study design

The overall study design was a cross-sectional survey among REC members and researchers conducted between November 2020 and January 2021. The protocol was developed as described below, and not published in advance. The questionnaire was designed specifically for this study.

### Questionnaire design

There is a considerable literature relating to different types of research design [9]. Based on this, and discussions among the authors and members of the European-funded EVBRES consortium, we identified 31 research methodologies commonly reviewed by RECs (see Table 1).

**Table 1:** List of research methodologies

**General category**	**Methodology**
Questionnaires	Nonintrusive questionnaire study
	Intrusive questionnaire study
	Validated clinical questionnaire study, e.g. with the possibility of being used to make a clinical diagnosis
Interviews	Nonintrusive interview telephone (audio only)
	Nonintrusive interview online (video and audio)
	Nonintrusive interview face to face
	Intrusive interview telephone (audio only)
	Intrusive interview online (video and audio)
	Intrusive interview face to face
Focus groups	Nonintrusive focus group remote (video conferencing)
	Nonintrusive focus group face to face
	Intrusive focus group remote (video conferencing)
	Intrusive focus group face to face
Intervention studies	Minor psychological or behavioural intervention study, e.g. subtle (designed to be unnoticed) changes to surroundings or ways information is presented or services delivered
	Major psychological or behavioural intervention study, e.g. overt changes to surroundings or how information is presented or services delivered
	Clinical psychology/psychiatry intervention study, e.g. involving the care of participants with diagnosed mental health conditions
	Physiological intervention study, e.g. different exercise regimens
Clinical/drug studies	Randomised nondrug clinical study (e.g. different patient groups assigned to different therapies)
	Phase I clinical trial (“first-in-man” administration of a new drug compound to around 20 people to test safety)
	Phase II clinical trial (to determine if the drug works (efficacy), usually in about 200 people)
	Phase III clinical trial (larger test of efficacy and acceptability, usually in about 2000 people)
	Phase IV clinical trial (post-marketing studies, usually long term once drug is being prescribed/used regularly)
Genetic research	Whole genome sequencing (where the whole genetic code unique to an individual will be determined)
	Genetic testing (small number of genes/markers) with no clinical significance (i.e. related to hair colour, general exercise performance)
	Genetic testing (small number of genes/markers) with clinical significance (e.g. related to potential/current diseases)
Observational studies	Observational study in public spaces, e.g. train stations, in parks
	Observational study in private space, e.g. in hospital wards, classrooms
Data studies	Anonymous secondary data analysis (analysing previously collected data sets without being able to identify who the data comes from)
	Identifiable secondary data analysis (analysing previously collected data sets and being able to identify who the data comes from)
	Anonymous secondary analysis of healthcare data
	Identifiable secondary analysis of healthcare data

The working group agreed a definition of risk that focussed specifically on research participants:

The likelihood and subsequent effect of physical, psychological, social or other harms on the research participant.

And it was noted that although RECs normally look at risk in the context of benefits and safeguards, this questionnaire was to focus specifically on risks that may come directly from the research methodology. This was described in the questionnaire by the statement:

We acknowledge that ethics committees/IRBs (and others) often weigh the acceptability of risk in light of potential benefits. However, in this survey, we are specifically trying to understand the contribution of research design types to overall risk assessments.

The questionnaire opened with a number of demographic questions, including three questions probing whether concepts of anonymity or consent can be viewed as mitigations for risks (see Supplementary information for wording of questions).

Following the demographic questions, the 31 research methodologies were presented alongside a 10-point Likert scale from “1: Not At All Risky” to “10:  Extremely Risky”. The 31 methodologies were presented as groups of related methodologies under the headings in Table 1. Identical instructions were included on each page:

On a scale of 1 (not at all risky) to 10 (extremely risky) what level of risk do you think is generally characteristic of the following types of research design?

As a number of study types included the word “intrusive” defined for participants as follows:

We will use the word ‘intrusive’ to mean research exploring significant factors affecting the participant (or their family's or community's) health, well-being and security (financial, physical etc.).

And a reminder of this definition was placed on every page where the word intrusive was used.

After the questions on study methodologies, a final open-text question was added to gather qualitative data and allow participants to express any other views they might hold:

Finally, in designing this survey, we appreciate that risk is often very context dependent and linked to the potential benefits of the study being evaluated. However, the aim of this survey has been to try to quantify how the type of research design, in broad and general terms, contributes to the understanding of overall risks to research participants. If you would like to make any additional comments in relation to this survey or the topic of risk please do so below (optional).

The full questionnaire can be found in the Supplementary information.

### Ethics, hosting, recruitment and dissemination

The research design and questionnaire were reviewed and given a favourable opinion by the University of Portsmouth (UK) Science and Health Faculty Ethics Committee (review number: SHFEC 2020-78). It was subsequently hosted on the Jisc survey platform (formerly Bristol Online Survey) [10] and open between 12th November 2020 and 22nd January 2021. Links to the survey were disseminated to the contacts listed in Table 2 by email (see Supplementary information), with the request to pass the survey on to anyone else that respondents thought might be interested (snow-ball sampling).

**Table 2:** Initial dissemination list

**Country**	**Contact**
UK	Chairs of RECs at the University of Portsmouth
UK	Members of the UK Ministry of Defence research ethics committee
UK	Members of the Public Health England research ethics committee (now known as the UK Health Security Agencies Research Ethics and Governance group UKHSA REGG).
UK	Members of the UK’s Health Research Authority (HRA) Hampshire A REC
UK	Members of the HRA Confidentiality Advisory Group (CAG)
Australia	The email list of the Australian Health Research Ethics Committee network
UK/international	The UK Health Research Authority communications team, asking them to circulate among members
EU/international	Various contacts at EUREC (a EU ethics committee organisation) and within the European Commission’s ethics directorate with a request to circulate among email lists
International	Other contacts known to the research team

As this was an anonymous survey, no explicit participant information sheet or consent form was used, however, a brief statement as to the purpose of the survey and how to find out more information was included on the first page of the survey, followed by a consent item, and then a brief thank you and reminder of the link to the overall project were added at the end (see Supplementary information).

### Data analysis

Descriptive statistics were used to describe the quantitative data from the questionnaire. Demographic questions and questions on anonymity and consent were summarised using frequencies and percentages. Responses for ranking of each of the 31 research methodologies were summarised using frequencies, percentages and means. Using the mean score for each of the 31 research methodologies, a hierarchy of research designs based on risk relating to each research methodology was created. Kendall rank correlation applying a *P*-value of 0.05 was used to investigate agreement or concordance in ranking of each research methodology by role (researcher, research ethics committee member, both, neither) and geographic area of employment.

The qualitative data from the open-text question was coded independently by two researchers to identify and agree on themes. The number of comments coded to each theme was presented numerically, while the content of the comments was used to contextualise the quantitative data. The raw data, coded to themes, has been included in the Supplementary materials.

## Results

**Fig. 1:**
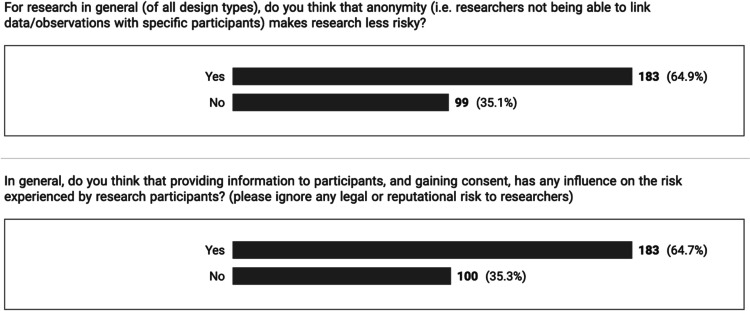
Relation of anonymity and consent to perceptions of risk

Two-hundred and eighty-three responses were received for the survey from respondents described in Fig. 1, located mostly in the UK (51%), Australia or New Zealand (29%) and the EU (14%). There was an almost identical one-third to two-third split between respondents who thought that either anonymity or consent made a contribution to risk (Table 3 and Fig. 1).

**Table 3:** Characteristics of survey respondents

***Demographic question***	***Yes***	***No***
Do you hold a higher research degree (e.g. MA, MSc, PhD, MD?)	227 (80.2%)	56 (19.8%)
Are you a registered clinician?	86 (30.4%)	197 (69.6%)
Is conducting research explicitly in your job title?	144 (50.9%)	139 (49.1%)
Have you ever been listed as an author on a paper in a peer-reviewed journal?	229 (80.9%)	54 (19.1%)
**Would you consider yourself as a researcher, research ethics committee member, both or neither?**		
Researcher	48 (17%)	
Research ethics committee member	96 (33.9%)	
Both	126 (44.5%)	
Neither	13 (4.6%)	
**How much professional research experience do you have?**		
Up to 5 years	40 (14.1%)	
From 5 to 10 years	38 (13.4%)	
From 10 to 20 years	65 (23%)	
More than 20 years	98 (34.6%)	
Not applicable	42 (14.8%)	
**In which area is your main employer registered?**		
Africa	0 (0%)	
Australia or New Zealand	82 (29.3%)	
Mexico, Central or South America	2 (0.7%)	
European Union	40 (14.3%)	
Middle East	0 (0%)	
Other European (non-EU) country	10 (3.6%)	
UK	143 (51.1%	
USA or Canada	3 (1.1%)	

### Risks based on methodology

The Likert scales provided a 10-point distribution for each of the 31 study types describing respondents’ perception of risk. The responses are summarised in Table 4. Statistical analysis demonstrated consistent, shared views in the ranking between role and geographic area of employment (see Supplementary information).

**Table 4:**
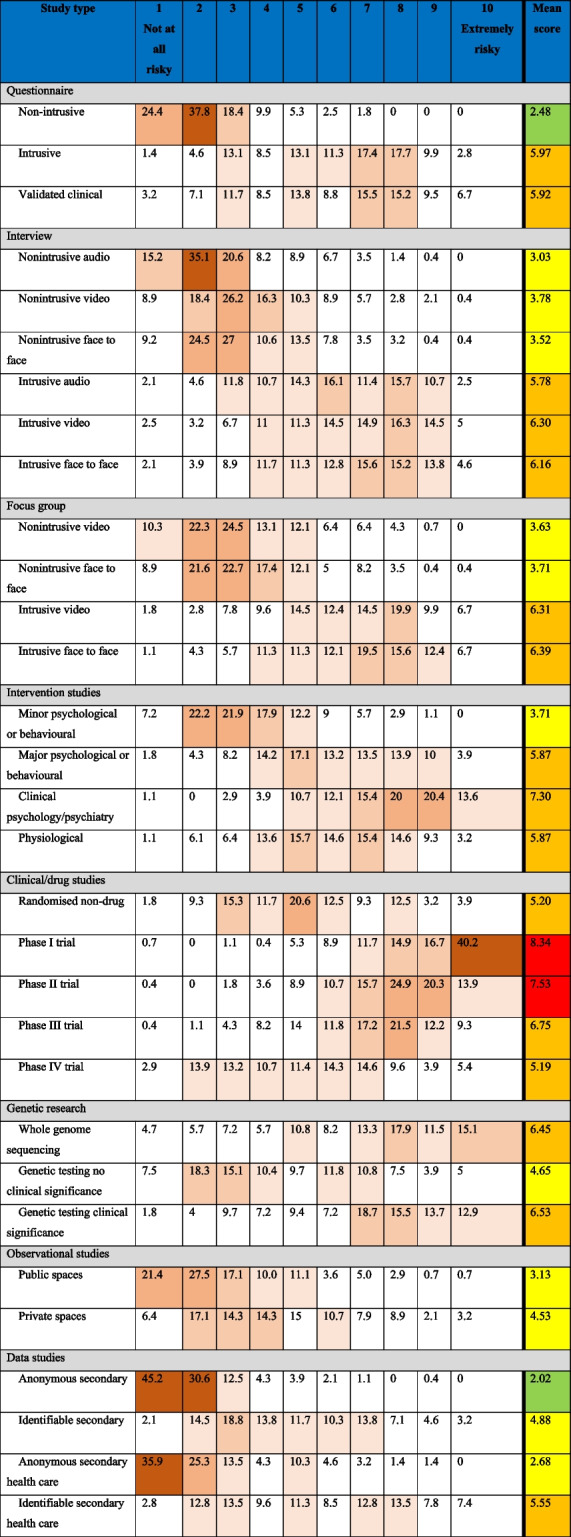
Risk scores for the different research methodologies. Following study name, columns 1 through 10 indicate percentage of respondents assigning each score. Brown > 30%, dark red > 20%, pink > 15% and rose > 10%. The final column shows overall mean score (scale 1 to 10) with green < 2.5 lowest risk, yellow between 2.5 and 5 low risk, orange between 5 and 7.5 high risk, red between 7.5 and 10 highest risk

**Fig. 2:**
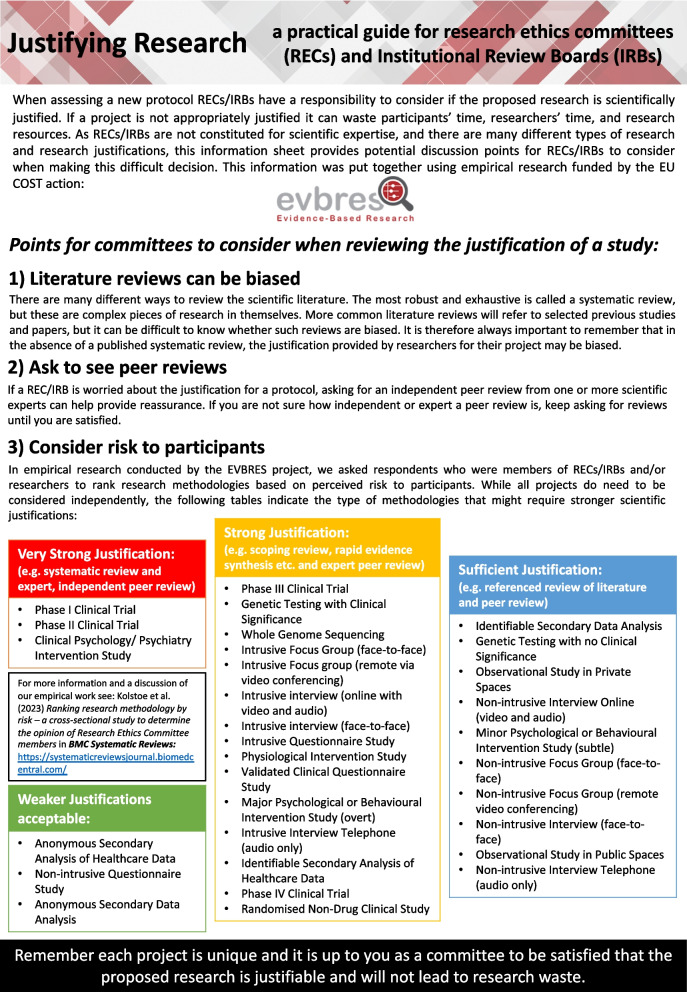
Information sheet for research ethics committees

Guided by this empirically derived hierarchy of risk, we then designed a one-page information sheet for use by REC members when conducting reviews (Fig. 2). Following introductory comments warning about bias in research literature, the option to seek additional peer review and a reminder that considering risk to participants is a central role of ethics review, we highlighted which research methodology fell into which of the four risk levels so as to inform REC conversations relating to study justification.

### Qualitative data

Eighty-seven free text comments were made. The text was coded by two investigators who discussed and agreed themes, as presented in Table 5.

**Table 5:** Main and subthemes derived from the qualitative, open-text question

**Main category**	**Subcategory**	**Number of references**	**Illustrative statement**
**Comment on the survey**		39	“Well presented and structured survey”
	Extra question or options needed	8	“I'm a retired clinician - nowhere to say this in the survey”
	Questions not specific enough	10	“Unable to make any nuanced response with categorical choices”
	Thanks, appreciation, offer of help	9	“Glad you are examining these matters”
**Comment on specific study designs**		7	“The survey should study research where non consenting bystanders may be unwittingly involved and privacy compromised and research that involves facial recognition”
	Broader methodology concerns	5	“I consider poor study design likely to cause biased findings to be a significant risk”
	Conduct of researchers	7	“I think it depends on the intent and conduct of the researchers”
	Clinical trials	2	“I have greater experience of clinical studies and trials and I am aware this affects my perception of risks when NHS treatment is involved”
	Observational study	2	“If you are running a covert observational study in public that has different risks associated in comparison to a clinical controlled environment”
	Surveys	1	“Our committee is seeing increasing numbers of studies where psychological screening tools, for eg anxiety and depression, are being used in anonymous online questionnaires. If they were being used in a face to face setting by a qualified practitioner, there are scores that would indicate that the person concerned should be urged to see further help, even though they do not provide a clinical diagnosis…”
**Comments on risk and benefit**		25	“Risk is not directly linked to potential benefits”
**Consent**		3	“Consent becomes highly apposite in the context of full and understandable explanations of potential risk; we are all entitled to choose our risk level provided we are properly informed”
**Context**		25	“All of the above answers depend on the context, the participants, researchers experience and the potential benefit of the research”
**Data & privacy**		7	“There is a relationship in many people eyes between privacy and risk which I have factored into my answers”
	Anonymity	4	“I think ensuring participant anonymity wherever possible is a very important element of good ethical practice”
	Confidentiality	1	“In my view, weaknesses in study design and in particular uncertainty about the risk that a study may not have enough power to reach a valid conclusion in the public interest, are quite often hard to weigh against the risk of disclosing personal information that could be objectionable to some individuals”
**PPI (patient & public involvement)**		2	“I was hoping that participatory designs might have been included in the study as this affected how I answered the earlier question about risk in all kinds of design. When studies can include participants in their design then this empowerment/opportunity for voice can reduce the risk”
**Reference to national guidance**		1	“Determination of risk in part is guided by the Australian National Health and Medical Research standards. However, risk must be determined on more than methodology. Inappropriate data management plans and publication can make low risk methodological studies into ethical nightmares. Applications must be reviewed on a case by case basis”

## Discussion

The three types of studies with the highest perceived level of risk with mean scores above 7.5 in our 10-point Likert scale were the Phase I and II clinical trials and clinical psychology/psychiatry intervention studies. If strength of study justification is to be linked to perceptions of risk, these would be the types of studies that require the highest level of scientific evidence in the form of a systematic review. However, if, as is often the case, a systematic review cannot be referenced, it may be problematic for a REC to either reject the study outright or base their favourable opinion on the production of a systematic review (among any other requests relating to recruitment, etc.). As such, while our work indicates that RECs should be careful in the absence of a systematic review for these types of studies, they should instead seek pragmatic alternatives such as requiring evidence of a robust, independent, peer review. However, even this compromise can sometimes be difficult in commercially sensitive Phase 1 trials where sponsors and contract research organisation are reticent to share commercially sensitive protocols. Here, the solution might be asking to see review by expert regulators such as the UK’s Medicines and Healthcare products Regulatory Agency (MHRA) or US’s Food and Drug Administration (FDA), which are often legally required in parallel to the REC review. Regardless of the exact solution, our results indicate that RECs should in general seek a high level of justification for these three types of studies.

**Fig. 3:**
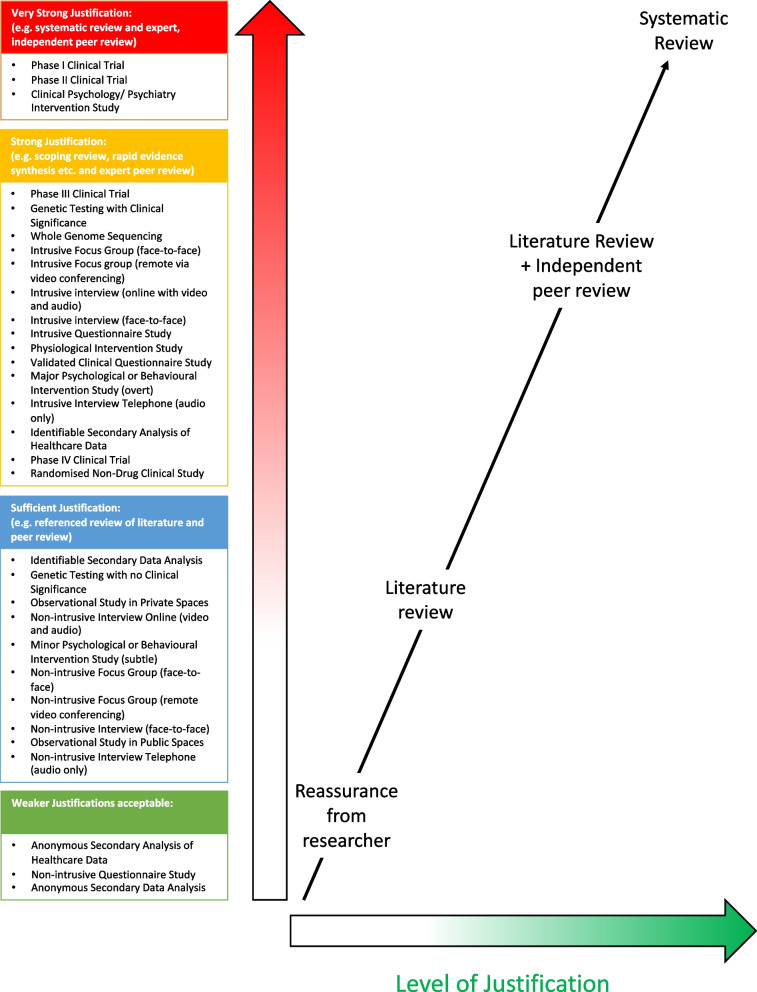
A proposal for mapping levels of risk from research methodology onto levels of justification/reviews

Fifteen different study methodologies were included in the second group (with a risk score above 5, i.e. the middle of our Likert scale), where a systematic review would be ideal, but other types of review may suffice. This raises the interesting question as to the difference between review methodologies. The literature in this area is often conflicting with attempts made to define multiple different review methodologies such as scoping reviews, umbrella reviews, rapid reviews, narrative reviews and meta-analysis. It is beyond the scope of this project to also propose a hierarchy of review types, but it might be possible to envisage a model where types of reviews were approximately mapped onto level of risk (Fig. 3). In our figure, we suggest that the minimum level of justification may be taking the researcher’s word that the project is worthwhile, the maximum being a full systematic review and then different other types of review coupled (or not) with independent peer review occupying the intervening space.

We mapped the remaining thirteen study types that respondents to our survey ranked as requiring lower levels of justification onto Fig. 3. This is not to say that a systematic, or other, type of review is not needed for justifying such lower-risk studies but rather that the REC may, a priori, be content with a less robust justification for this sort of study methodology. Of course, other aspects of the study such as vulnerability of the participants or other contextual issues might mean that the REC will still want to see a robust justification even with a lower-risk study methodology, but we feel that this figure could still be helpful to guide committee deliberations.

Obtaining a sample from a population of ethics committee members with experience in the critical examination of research protocols was particularly valuable with respect to commenting on both the value of our hypothesis (that methodology influences perceptions of risk) and our own survey/questionnaire design. Of the 87 free text comments, we were pleased that nine provided positive feedback on our work. Of the thirty other comments in relation specifically to our work, one particular theme with ten comments is related to the questions, or at least definitions of the different research methodologies, not being specific enough. This is not surprising as REC members are well aware that although “rules of thumb” can be helpful, the context of each and every project reviewed by a REC is vital for coming to decisions. ‘Hence, we broadly agree with the comment from one respondent:

I missed the opportunity to say 'sometimes' rather than yes or no on some of the questions. A number of times my response would have been 'it depends' - as it is I think the questionnaire will give only a broad brush and rather simplistic analysis of risk appreciation related to research participants.

In addition, when compiling the list of thirty-one study methodologies, we inadvertently missed out “human challenge studies”, a type of design that was subsequently widely discussed due to the COVID-19 pandemic [11]. While it would have been interesting to have included this as a study type in our survey, it should be noted that the types of methodologies used for human challenge studies are broadly covered in the thirty-one categories already included, albeit not the aspect of deliberately exposing healthy volunteers to a pathogen of interest.

Two topics that frequently occupy RECs are arrangements for consenting participants, and treating data anonymously. We therefore added two questions to determine whether these aspects affected perceptions of risk. Interestingly, in both cases, approximately two-thirds of respondents felt that treating data anonymously, and ensuring participants are provided with sufficient information and the opportunity to consent, reduced risk. Such risk could not be to physical harm (as both processes are essentially administrative), so the results indicate that respondents are also viewing risk to participants in relation to social concepts such as privacy and perhaps rights to self-determination. This observation provides strong evidence that alongside the level of scientific/academic justification, RECs should also pay closer attention to issues linked to anonymity and the consenting process for higher-risk studies. Indeed, to a certain extent, this already happens, as when considering the hierarchy of methodologies, some of the lowest risk study designs are anonymous or not always able to provide information or seek consent from participants (e.g. secondary analysis of healthcare data or public observation studies). Similarly, high levels of information coupled with exhaustive consent processes are more often found in the higher-risk studies such as Phase I clinical trials.

## Conclusions

In conducting this work we are not seeking to provide concrete guidance for RECs, but rather highlight the observation that research methodology does impact how REC members (and others) perceive the risk of research. While it would be a mistake for RECs to always demand the type of review we suggest, we hope that our guidance will help RECs decide whether the evidence for a study has been reviewed in an appropriately systematic way. 

### Supplementary Information

**Additional file 1.** Tables: Demographic and questions on Anonymity and Consent. Questions on Research Methodologies. Dissemination email. Participant Information and Consent. Statistical analysis of ranking by role. Statistical analysis of ranking by geographic area of employment.

**Additional file 2.** Qualitative data: Anonymity. Broader Methodology Concerns. Clinical Trials. Codebook - EVBRES Qu20. Comment on our Survey. Comment on specific study designs. Comments on Risk and Benefit. Conduct of researchers. Confidentiality. Consent. Context. Data & Privacy. Extra question or options needed. Observational Study. PPIE. Questions not specific enough. Reference to national guidance. Response to Reviewers ver2. Surveys. Thanks, appreciation, offer of help.

## Data Availability

The datasets used and/or analysed during the current study are available from the corresponding author on reasonable request.
