# Supplementary material for: Ranking Research Methodology by Risk — a cross-sectional study to determine the opinion of research ethics committee members
Source: Syst Rev. 2023 Sep 1;12:154. doi: 10.1186/s13643-023-02295-1 (PMC10472668; doi:10.1186/s13643-023-02295-1)
Supplement: Supplementary file 1 — Additional file 1. Tables: Demographic and questions on Anonymity and Consent. Questions on Research Methodologies. Dissemination email. Participant Information and Consent. Statistical analysis of ranking by role. Statistical analysis of ranking by geographic area of employment. [file 13643_2023_2295_MOESM1_ESM.docx]

**Supplementary Information**

***Demographic and questions on Anonymity and Consent***

| **Question** | **Answer** |
| --- | --- |
| Do you hold a higher research degree (e.g. MA, MSc, PhD, MD etc.)? | Yes  No |
| Are you a registered clinician? | Yes  No |
| Would you consider yourself as a researcher, research ethics committee (IRB) member, both or neither? | Researcher  Research Ethics Committee Member  Both  Neither |
| Have you ever been listed as an author on a paper in a peer reviewed journal? | Yes  No |
| Is conducting research explicitly in your current job description? | Yes  No |
| How much professional research experience do you have? | Up to 5 years  From 5 to 10 years  From 10 to 20 years  More than 20 years  Not Applicable |
| In which area is your main employer registered? | Africa  Australia or New Zealand  Asia  Mexico, Central or South America  European Union  Middle East  Other European (non EU) country  United Kingdom  United States or Canada |
| **Anonymous vs Identifiable Research** | |
| In general, for research involving just data, do you think that anonymity (i.e. researchers not being able to link data with specific participants) makes research less risky? | Yes  No |
| For research in general (of all design types), do you think that anonymity (i.e. researchers not being able to link data/observations with specific participants) makes research less risky? | Yes  No |
| **Consent** | |
| In general, do you think that providing information to participants, and gaining consent, has any influence on the risk experienced by research participants? (please ignore any legal or reputational risk to researchers) | Yes  No |

***Questions on Research Methodologies***

| ***Question*** | ***Responses*** |
| --- | --- |
| *On a scale of 1 (not at all risky) to 10 (extremely risky) what level of risk do you think is generally characteristic of the following types of research design?* | |
| Non-intrusive questionnaire study | Likert Scale |
| Intrusive questionnaire study | Likert Scale |
| Validated clinical questionnaire study e.g. with the possibility of being used to make a clinical diagnosis | Likert Scale |
| Non-intrusive interview telephone (audio only) | Likert Scale |
| Non-intrusive interview online (video and audio) | Likert Scale |
| Non-intrusive interview face to face | Likert Scale |
| Intrusive interview telephone (audio only) | Likert Scale |
| Intrusive interview online (video and audio) | Likert Scale |
| Intrusive interview face to face | Likert Scale |
| Non-intrusive Focus group remote (video conferencing) | Likert Scale |
| Non-intrusive Focus group face to face | Likert Scale |
| Intrusive Focus group remote (video conferencing) | Likert Scale |
| Intrusive Focus group face to face | Likert Scale |
| Minor psychological or behavioural intervention study e.g. subtle (designed to be unnoticed) changes to surroundings or ways information is presented, or services delivered | Likert Scale |
| Major psychological or behavioural intervention study e.g. overt changes to surroundings or how information is presented, or services delivered | Likert Scale |
| Clinical Psychology/Psychiatry intervention study e.g. involving the care of participants with diagnosed mental health conditions | Likert Scale |
| Physiological intervention study e.g. different exercise regimens | Likert Scale |
| Randomised non-drug clinical study (e.g. different patient groups assigned to different therapies) | Likert Scale |
| Phase I Clinical Trial ("First in man" administration of a new drug compound to around 20 people to test safety) | Likert Scale |
| Phase II Clinical Trial (To determine if the drug works (efficacy), usually in about 200 people) | Likert Scale |
| Phase III Clinical Trial (Larger test of efficacy and acceptability, usually in about 2000 people) | Likert Scale |
| Phase IV Clinical Trial (Post-marketing studies, usually long term once drug is being prescribed/used regularly) | Likert Scale |
| Whole genome sequencing (where the whole genetic code unique to an individual will be determined) | Likert Scale |
| Genetic testing (small number of genes/markers) with no clinical significance (i.e. related to hair colour, general exercise performance etc.) | Likert Scale |
| Genetic testing (small number of genes/markers) with clinical significance (e.g. related to potential/current diseases) | Likert Scale |
| Observational study in public spaces e.g. train stations, in parks etc. | Likert Scale |
| Observational study in private space e.g. in hospital wards, classrooms | Likert Scale |
| Anonymous secondary data analysis (analysing previously collected data sets without being able to identify who the data comes from) | Likert Scale |
| Identifiable secondary data analysis (analysing previously collected data sets and being able to identify who the data comes from) | Likert Scale |
| Anonymous secondary analysis of healthcare data | Likert Scale |
| Identifiable secondary analysis of healthcare data | Likert Scale |
| **Final Qualitative question** | |
| Finally, in designing this survey we appreciate that risk is often very context dependent and linked to the potential benefits of the study being evaluated. However, the aim of this survey has been to try to quantify how the type of research design, in broad and general terms, contributes to the understanding of overall risks to research participants. If you would like to make any additional comments in relation to this survey or the topic of risk please do so below (optional). | *Open Text* |

***Dissemination email***

| Dear all,  As part of a European project (https://evbres.eu) I am conducting a piece of research looking into how research ethics committee members understand risk in relation to research design.  Would it be possible to circulate the attached link to an anonymous, 10 minute, questionnaire to your members and anyone else who might have an interest?  The survey has a favourable opinion from the Science & Health Faculty Ethics committee at the University of Portsmouth (UK) ref. SHFEC 2020-78 to use a snowball recruitment strategy and email addresses gathered from public websites. It is being distributed internationally.  https://portsmouth.onlinesurveys.ac.uk/evbres-rec-survey  Thanks! |
| --- |

***Participant Information and Consent***

| ***Participant Information*** |
| --- |
| EVBRES is an EU COST funded grant with the aim of reducing research waste by promoting better use of evidence used for the justification of new research projects.  This questionnaire has been created to gain an understanding of how researchers and ethics committee members rate the risks inherent in various research design types.  All responses to this questionnaire are anonymous, no IP addresses or other tracking information is being collected, and therefore your response cannot be identified or withdrawn once submitted.  The results of this questionnaire will be analysed by the EVBRES team and used as part of a larger project to create guidance for research ethics committees and others who evaluate research. If you would like any further information please email Simon Kolstoe on simon.kolstoe@port.ac.uk or see https://evbres.eu  The survey was reviewed and received a favourable opinion from the Faculty of Science and Health Research Ethics Committee at the University of Portsmouth (UK), reference: SHFEC 2020-78 |
| ***Consent Clause:*** |
| By completing this questionnaire you are providing consent for us to use your anonymously provided data for the purpose described above. Please tick "agree" if you wish to continue, otherwise please close the browser window. |
| ***Closing Thanks and Information*** |
| Thank you for taking part in our research.  The results of this questionnaire will be analysed by the EVBRES team and used as part of a project to create guidance for research ethics committees and others who evaluate research. If you would like any further information, or a copy of the results, please email Simon Kolstoe on simon.kolstoe@port.ac.uk or see https://evbres.eu |

***Statistical analysis of ranking by role***

|  | **All Participants** | **Researcher** | **Research Ethics Committee Member** | **Both** | **Neither** |
| --- | --- | --- | --- | --- | --- |
| **All Participants** | ***―*** | .901^**^ | .931^**^ | .961^**^ | .897^**^ |
| **Researcher** | .901^**^ | ***―*** | .849^**^ | .880^**^ | .867^**^ |
| **Research Ethics Committee Member** | .931^**^ | .849^**^ | ***―*** | .892^**^ | .862^**^ |
| **Both** | .961^**^ | .880^**^ | .892^**^ | ***―*** | .867^**^ |
| **Neither** | .897^**^ | .867^**^ | .862^**^ | .867^**^ | 1.000 |
| *** Correlation is significant at the 0.01 level* | | | | | |

***Statistical analysis of ranking by geographic area of employment***

|  | **Australia/**  **New Zealand** | **European Union** | **Europe,**  **Non-**  **European**  **Union** | **Mexico/**  **Central America/ South America** | **Canada/**  **United States** | **United Kingdom** |
| --- | --- | --- | --- | --- | --- | --- |
| **Australia/**  **New Zealand** | ***―*** | .880** | .841** | .454** | .755** | .914** |
| **European**  **Union** | .880** | ― | .892** | .437** | .772** | .880** |
| **Europe,**  **Non-**  **European**  **Union** | .841** | .892** | ― | .424** | .751** | .858** |
| **Mexico/**  **Central America/**  **South America** | .454** | .437** | .424** | ― | .441** | .411** |
| **Canada/**  **United States** | .755** | .772** | .751** | .441** | ― | .781** |
| **United**  **Kingdom** | .914** | .880** | .858** | .411** | .781** | ― |
| ***Correlation is significant at the 0.01 level* | | | | | | |
