# Supplementary material for: Ranking Research Methodology by Risk — a cross-sectional study to determine the opinion of research ethics committee members
Source: Syst Rev. 2023 Sep 1;12:154. doi: 10.1186/s13643-023-02295-1 (PMC10472668; doi:10.1186/s13643-023-02295-1)
Supplement: Supplementary file 2 — Additional file 2. Qualitative data: Anonymity. Broader Methodology Concerns. Clinical Trials. Codebook - EVBRES Qu20. Comment on our Survey. Comment on specific study designs. Comments on Risk and Benefit. Conduct of researchers. Confidentiality. Consent. Context. Data & Privacy. Extra question or options needed. Observational Study. PPIE. Questions not specific enough. Reference to national guidance. Response to Reviewers ver2. Surveys. Thanks, appreciation, offer of help. [file 13643_2023_2295_MOESM2_ESM.zip › AnonymityR1.docx]

Files\\EVBRES Qu20 - § 4 references coded [ 4.49% Coverage]

Reference 1 - 1.12% Coverage

The questions about anonymised data assume that the anonymisation is reliable and irreversible, which is not always true.

Reference 2 - 1.12% Coverage

I think ensuring participant anonymity wherever possible is a very important element of good ethical practice.

Reference 3 - 1.12% Coverage

Being able to re-identify participants is important to enable data verification. I am not in favour of anonymisation of research data except in exceptional circumstances when the risks associated with identifying individual participants are very high.

Reference 4 - 1.12% Coverage

Anonymity is commonly over-represented as the solution to risk, especially in big data analysis. This is a poorly understood over-simplification of the range of risks which relate to how human beings behave and what they might do with data and any analytical findings.
