# Supplementary material for: Ranking Research Methodology by Risk — a cross-sectional study to determine the opinion of research ethics committee members
Source: Syst Rev. 2023 Sep 1;12:154. doi: 10.1186/s13643-023-02295-1 (PMC10472668; doi:10.1186/s13643-023-02295-1)
Supplement: Supplementary file 2 — Additional file 2. Qualitative data: Anonymity. Broader Methodology Concerns. Clinical Trials. Codebook - EVBRES Qu20. Comment on our Survey. Comment on specific study designs. Comments on Risk and Benefit. Conduct of researchers. Confidentiality. Consent. Context. Data & Privacy. Extra question or options needed. Observational Study. PPIE. Questions not specific enough. Reference to national guidance. Response to Reviewers ver2. Surveys. Thanks, appreciation, offer of help. [file 13643_2023_2295_MOESM2_ESM.zip › Broader Methodology ConcernsR1.docx]

Files\\EVBRES Qu20 - § 5 references coded [ 5.09% Coverage]

Reference 1 - 1.12% Coverage

Risk is not always direct- studies of any design can be damaged by faulty data collection and analysis and if then used to inform practice or policy,participants as well as the wider public are at risk. I see far too much proposed analysis that is seriuously flawed and it would be better if the studies concerned were not allowed to start in the first place unless their analytical element is strongly challenged and corrected beforehand as it is the consequences of a flawed conclusion that presents the greatest risk by far

Reference 2 - 1.12% Coverage

It all depends on the way the research team handle the participants and in the next phase the data.

Reference 3 - 1.12% Coverage

I prefer a generally small risk in research instead of higher risk of exposing people to untested interventions i practice. Well planned research is in general less risky than experiments in practice outside research settings.

Reference 4 - 1.12% Coverage

I would add randomization as potential factor amplifying risks, e.g. randomization in surgical research. Also a distinction between invasive/non-invasive research is important.

Reference 5 - 0.59% Coverage

I consider poor study design likely to cause biased findings to be a significant risk.
