# Supplementary material for: Ranking Research Methodology by Risk — a cross-sectional study to determine the opinion of research ethics committee members
Source: Syst Rev. 2023 Sep 1;12:154. doi: 10.1186/s13643-023-02295-1 (PMC10472668; doi:10.1186/s13643-023-02295-1)
Supplement: Supplementary file 2 — Additional file 2. Qualitative data: Anonymity. Broader Methodology Concerns. Clinical Trials. Codebook - EVBRES Qu20. Comment on our Survey. Comment on specific study designs. Comments on Risk and Benefit. Conduct of researchers. Confidentiality. Consent. Context. Data & Privacy. Extra question or options needed. Observational Study. PPIE. Questions not specific enough. Reference to national guidance. Response to Reviewers ver2. Surveys. Thanks, appreciation, offer of help. [file 13643_2023_2295_MOESM2_ESM.zip › Clinical TrialsR1.docx]

Files\\EVBRES Qu20 - § 2 references coded [ 1.96% Coverage]

Reference 1 - 0.84% Coverage

For the clinical trials questions, I don’t think the particular phase I,II, III changes the risk to the individual when there is a larger group of participants. After the initial phases and move into what was described as stage IV, the risk may reduce as the research study will make the distinction between efficacy and effectiveness. Efficacy is under ideal conditions with health research participants. Effectiveness is under real world conditions, with participants having potential health issues. The company may take more care in how they release their ’intervention’, i.e. I feel that the marketing and potential legal issues will help to further reduce risks.

Reference 2 - 1.12% Coverage

I have greater experience of clinical studies and trials and i am aware this affects my perception of risks when NHS treatment is involved
