# Supplementary material for: Ranking Research Methodology by Risk — a cross-sectional study to determine the opinion of research ethics committee members
Source: Syst Rev. 2023 Sep 1;12:154. doi: 10.1186/s13643-023-02295-1 (PMC10472668; doi:10.1186/s13643-023-02295-1)
Supplement: Supplementary file 2 — Additional file 2. Qualitative data: Anonymity. Broader Methodology Concerns. Clinical Trials. Codebook - EVBRES Qu20. Comment on our Survey. Comment on specific study designs. Comments on Risk and Benefit. Conduct of researchers. Confidentiality. Consent. Context. Data & Privacy. Extra question or options needed. Observational Study. PPIE. Questions not specific enough. Reference to national guidance. Response to Reviewers ver2. Surveys. Thanks, appreciation, offer of help. [file 13643_2023_2295_MOESM2_ESM.zip › Codebook - EVBRES Qu20 - 27 Jun 2023R1.docx]

EVBRES Qu20

## Nodes

| **Name** | **Description** | **Files** | **References** |
| --- | --- | --- | --- |
| Comment on our Survey |  | 1 | 39 |
| Extra question or options needed |  | 1 | 8 |
| Questions not specific enough |  | 1 | 10 |
| Thanks, appreciation, offer of help |  | 1 | 9 |
| Comment on specific study designs |  | 1 | 7 |
| Broader Methodology Concerns |  | 1 | 5 |
| Conduct of researchers |  | 1 | 7 |
| Clinical Trials |  | 1 | 2 |
| Observational Study |  | 1 | 2 |
| Surveys |  | 1 | 1 |
| Comments on Risk and Benefit |  | 1 | 25 |
| Consent |  | 1 | 3 |
| Context |  | 1 | 25 |
| Data & Privacy |  | 1 | 7 |
| Anonymity |  | 1 | 4 |
| Confidentiality |  | 1 | 1 |
| PPIE |  | 1 | 2 |
| Reference to national guidance |  | 1 | 1 |
