# Supplementary material for: Ranking Research Methodology by Risk — a cross-sectional study to determine the opinion of research ethics committee members
Source: Syst Rev. 2023 Sep 1;12:154. doi: 10.1186/s13643-023-02295-1 (PMC10472668; doi:10.1186/s13643-023-02295-1)
Supplement: Supplementary file 2 — Additional file 2. Qualitative data: Anonymity. Broader Methodology Concerns. Clinical Trials. Codebook - EVBRES Qu20. Comment on our Survey. Comment on specific study designs. Comments on Risk and Benefit. Conduct of researchers. Confidentiality. Consent. Context. Data & Privacy. Extra question or options needed. Observational Study. PPIE. Questions not specific enough. Reference to national guidance. Response to Reviewers ver2. Surveys. Thanks, appreciation, offer of help. [file 13643_2023_2295_MOESM2_ESM.zip › Comment on our SurveyR1.docx]

Files\\EVBRES Qu20 - § 39 references coded [ 38.22% Coverage]

Reference 1 - 1.12% Coverage

The questions about anonymised data assume that the anonymisation is reliable and irreversible, which is not always true.

Reference 2 - 1.12% Coverage

Slight flaw in that clinical research is not the only type of academic research. Maybe, there should have been an extra question: "Is your research experience entirely clinical, partly clinical or not at all clinical?"

Reference 3 - 1.12% Coverage

I’m a retired clinician - nowhere to say this in the survey. Did you involve research participants (or other PPI) in the design of the survey?

Reference 4 - 1.12% Coverage

I remember a paper linking perceptions of risk of research related to common everyday events. Do you have this? I could find the link if you want

Reference 5 - 1.12% Coverage

Thank you

Reference 6 - 1.12% Coverage

Some of these questions were difficult to answer because the examples given were so broad that it was effectively impossible to ascertain risk, e.g. F2F FG: the risk here depends very much on the participants themselves (i.e. their mental state, their inter-relationships, etc.) and the questions being asked. This necessitated guesstimating, rather than estimating - which is unlikely to lead to meaningful findings...

Reference 7 - 1.12% Coverage

Glad you are examining these matters

Reference 8 - 0.26% Coverage

I would be interested in the report, and would be particularly interested in the models of risk you are using (eg Andy Stirling?). Because within responses there will certainly be ambiguity - did a respondent interpret the question as being bland observation in a public place or looking at drug deals going down.

Reference 9 - 0.26% Coverage

This survey will give you a broad brush (and I expect some differences for example in how clinicians see risk especially around trials stages), but the really interesting issues may be in this borderland territory, where difficult risk judgements need to be made on the basis of the individual case in front of you.

Reference 10 - 1.12% Coverage

Well presented and structured survey

Reference 11 - 1.12% Coverage

The survey should study research where non consenting bystanders may be unwittingly involved and privacy compromised and research that involves facial recognition.

Reference 12 - 1.12% Coverage

I missed the opportunity to say ’sometimes’ rather than yes or no on some of the questions. A number of times my response would have been ’it depends’ - as it is I think the questionnaire will give only a broad brush and rather simplistic analysis of risk appreciation related to research participants

Reference 13 - 1.12% Coverage

Q4 - data only research - what does this mean? Q7 and Q12 - risk to whom, participant, researcher or both? Q10 , 1 - what therapies? the risk would vary so widely that the answer to this question without clarification is meaningless

Reference 14 - 1.12% Coverage

Some questions were a bit hard to answer - e.g. there would seem to be little point in collecting data without being able to identify participants, as it would severely limit the comparisons that could be made and the conclusions drawn.

Reference 15 - 1.12% Coverage

Unable to make any nuanced response with categorical choices

Reference 16 - 1.12% Coverage

No

Reference 17 - 0.27% Coverage

The survey is projecting a limited vision of the reality of research ethics committees as it is based on the assumption that the risks can be evaluated in abstracto.

Reference 18 - 0.64% Coverage

Your questionnaire does not provide the option to respond "I don’t know" or "not applicable" and thus creates a serious biais. The study falls under the growing trend of "empirical studies" in bioethics and one can wonder the nature of the conclusions you could draw from it. I would be happy to discuss this issue if you wish. Best regards, Dominique Sprumont Dominique.Sprumont@vd.ch

Reference 19 - 1.12% Coverage

study designs in and off themselves are not risky, they are appropriate depending on the research question. Risk is related to how a study is conducted. For that reason most of my responses were 5, neither high/low risk as a sensitive survey with very personal questions is not high risk if conducted correctly.

Reference 20 - 1.12% Coverage

Surprised that my answers assessed risk of face to face engagement, as greater than video, and telephone - risk of upset higher face to face..

Reference 21 - 1.12% Coverage

Easy to use on phone. Click and turn the page

Reference 22 - 1.12% Coverage

Thank you for this research. I look forward to reading the published articles upon completion.

Reference 23 - 1.12% Coverage

many questions felt abstract and there fore I tended to identify possible risk and rate risk factors high

Reference 24 - 1.12% Coverage

The survey does not allow for a retired option

Reference 25 - 1.12% Coverage

Time to complete survey should have been in information to participants (not just in email). Most risk tables not randomized in terms of risk levels. But a really useful survey - look forward to findings. The questions are highly relevant to our research ethics decision-making.

Reference 26 - 1.12% Coverage

Please note I am retired from paid employment but that doesn’t seem to be an option in the early questions. I indicated that I don’t conduct research but I have done so, until quite recently. These days, clinician includes allied health professionals such as nurses, pharmacists, dentists, physios etc. Was that your meaning?

Reference 27 - 1.12% Coverage

I think you sacrifice too much when trying to quantify risk to research participants in this manner. It could even hinder and misdirect. Sensitivity to the variables involved, such as context and content is incredibly important in developing a useful and grounded understanding. An illustration of reductionism such as this is risky.

Reference 28 - 0.16% Coverage

Risk is v Context dependant so answering these questions is almost like being blindfolded

Reference 29 - 0.26% Coverage

My answers have been given in comparison to the other research designs grouped together, not overall.

Reference 30 - 0.42% Coverage

This survey assumes someone is still employed. Many REC members are retired.

Reference 31 - 1.12% Coverage

As usual when discussing ethical issues, my response to most of these questions where you force a choice with relatively little information is ’it depends on the specific circumstances’ and so found this quite difficult.

Reference 32 - 1.12% Coverage

The expression used in one of the last questions before the graded ones asked about risk experienced by a person (and if informed consent affects it). Here subjective perception of risk and “objective” risk, or risk perceived by an outside observer can be very different. The survey does not really specify which is meant (I presume the latter), and this may lead to confusion.

Reference 33 - 1.12% Coverage

A definition of what is covered by the world ’risk’ would have helped in providing the answers.

Reference 34 - 1.12% Coverage

In the question about where is your employer based, followed by a list of countries, I am not employed so I did not answer that. An additional option may be useful in future studies.

Reference 35 - 1.12% Coverage

I wasn’t always sure how to interpret ’risky’ - but usually took this to mean ’risk of harm’, I think. Yes - many of my answers could have changed quite a bit if more parameters and design factors had been specified, e.g. chance of re-identifiability of anonymised data sets, sensitivity of topic in public observations, etc.

Reference 36 - 1.12% Coverage

Frustrating survey because risk is contextually driven. Observation in public spaces for example could include watching drug deals (higher risk) versus seeing how people interact with a public park (lower risk).

Reference 37 - 1.12% Coverage

You have not included in this questionnaire the risk perception of studies involving stem cells. I consider research involving the extraction of pluripotent and research administering artifically grown cells from stem cells currently to be at the highest risk level, thus a 10.

Reference 38 - 1.12% Coverage

N/A

Reference 39 - 1.12% Coverage

You didn’t mention if the audio and video interviews would be recorded and/or transcribed later. I see higher risk there as participant’s data could be accessed by others any other time and info could be stolen.
