# Supplementary material for: Ranking Research Methodology by Risk — a cross-sectional study to determine the opinion of research ethics committee members
Source: Syst Rev. 2023 Sep 1;12:154. doi: 10.1186/s13643-023-02295-1 (PMC10472668; doi:10.1186/s13643-023-02295-1)
Supplement: Supplementary file 2 — Additional file 2. Qualitative data: Anonymity. Broader Methodology Concerns. Clinical Trials. Codebook - EVBRES Qu20. Comment on our Survey. Comment on specific study designs. Comments on Risk and Benefit. Conduct of researchers. Confidentiality. Consent. Context. Data & Privacy. Extra question or options needed. Observational Study. PPIE. Questions not specific enough. Reference to national guidance. Response to Reviewers ver2. Surveys. Thanks, appreciation, offer of help. [file 13643_2023_2295_MOESM2_ESM.zip › Comment on specific study designsR1.docx]

Files\\EVBRES Qu20 - § 7 references coded [ 6.48% Coverage]

Reference 1 - 1.12% Coverage

Observational studies in public spaces can put research at risk if approached by a member of the public or police if researcher’s behaviour appears unusual and they may need to mitigate for this and have information about the study they can provide to reassure

Reference 2 - 1.12% Coverage

No difference in risk between audio, video, face to face studies (in general) Highest level of risk involving ’first in human’ (Phase I) studies (depending on study design eg staggered start) - plenty of examples in the literature of this (eg TGN1412 trial)

Reference 3 - 0.29% Coverage

What is faced by ethics committees is a constant balancing of risk ambiguity and uncertainty - will someone suffer psychological harm from having felt ’duped’ in a behavioural intervention study, will someone in a focus group on a low risk topic divulge something high risk about themselves, and will other participants keep that confidential. e.t.c.

Reference 4 - 1.12% Coverage

The survey should study research where non consenting bystanders may be unwittingly involved and privacy compromised and research that involves facial recognition.

Reference 5 - 1.12% Coverage

Genetic testing, AI advances, complicated regulatory environments (globalised), increase the risk of identification.

Reference 6 - 0.96% Coverage

Some questionnaires can raise v sensitive topics or highlight problems for which there are no solutions or generate new concerns. Phase 1 research into a ‘me too’ drug is likely to be less risky than a new class kid drug etc. Depends where & who is doing the research too , are other options available, what is the burden of the research commitment etc. Some questionnaire studies can pose a greater risk than taking a single additional blood sample for research. Who is determining what is considered a risk that is an individual decision.

Reference 7 - 0.74% Coverage

A phase 1 first in man is by definition riskier than a later phase of a trial. The risk is however different if this is a first in class or a variant of an exiting drug. Likewise interviews can seem quite risk free, however certain types of interviews can elicit strong emotional responses or reveal risks to the individual or others that the researcher has to have procedures to deal with. Observational studies, that seem very mild and low risk, can become high risk if they involve observation of behaviors in homeless drug users, for example. So although theoretically some designs are less "risky" than others, each has to be evaluated on its own merits.
