# Supplementary material for: Ranking Research Methodology by Risk — a cross-sectional study to determine the opinion of research ethics committee members
Source: Syst Rev. 2023 Sep 1;12:154. doi: 10.1186/s13643-023-02295-1 (PMC10472668; doi:10.1186/s13643-023-02295-1)
Supplement: Supplementary file 2 — Additional file 2. Qualitative data: Anonymity. Broader Methodology Concerns. Clinical Trials. Codebook - EVBRES Qu20. Comment on our Survey. Comment on specific study designs. Comments on Risk and Benefit. Conduct of researchers. Confidentiality. Consent. Context. Data & Privacy. Extra question or options needed. Observational Study. PPIE. Questions not specific enough. Reference to national guidance. Response to Reviewers ver2. Surveys. Thanks, appreciation, offer of help. [file 13643_2023_2295_MOESM2_ESM.zip › Comments on Risk and BenefitR1.docx]

Files\\EVBRES Qu20 - § 25 references coded [ 23.14% Coverage]

Reference 1 - 0.11% Coverage

Risk is not directly linked to potential benefits

Reference 2 - 1.01% Coverage

judging whether a level of risk is acceptable (or acceptable to offer participation to a person) is certainly linked to benefits. Who runs the risk and who is likely to receive the benefit is also highly relevant (they are not always the same person or societal group). The potential participant is certainly central to considering risks, but risks to the researcher, the sponsoring institution and people at large also have to be considered.

Reference 3 - 0.42% Coverage

For the clinical trials questions, I don’t think the particular phase I,II, III changes the risk to the individual when there is a larger group of participants. After the initial phases and move into what was described as stage IV, the risk may reduce as the research study will make the distinction between efficacy and effectiveness.

Reference 4 - 1.12% Coverage

I have greater experience of clinical studies and trials and i am aware this affects my perception of risks when NHS treatment is involved

Reference 5 - 1.12% Coverage

Although risk is quantifiable I do think individuals hold their own view of their risk spectrum. As a rec member I aim to take a broadly conservative approach - I know there may be downsides to this but I try to imagine myself as a relatively risk averse potential participant when considering risk in various studies. My thinking here is, if I take a cautious approach I am more likely to raise issues of risk for discussion that a less cautious person might be happy to take - almost like a minimum reasonable threshold. Not sure if I am explaining this well but hopefully that makes sense.

Reference 6 - 0.78% Coverage

Risk of stigmatising groups of people (eg indigenous peoples) must be taken into account. That is, identifying groups can be risky, not just identifying individuals.

Reference 7 - 0.32% Coverage

My conception of risk includes things that might not go to plan. eg observation in public space is generally low ish risk, but what if you observe criminal behaviour (or even if that’s what you are looking for). I would also say covert research high risk - including changing things people are not supposed to notice (seems benign, but I have seen applications where that was far from the case).

Reference 8 - 1.12% Coverage

A central problem is that people’s perception of risk can make a difference to the actual risk; and that complicates things, especially as our understanding of risk is generally very poor.

Reference 9 - 0.69% Coverage

I (as does the committee on which I serve) see asking people to reflect on their psychological wellbeing increases the risk that someone might self-harm. It is also likely that people who are concerned about their psychological well-being are more likely to answer this kind of survey, so not providing participants with an indication of what kind of responses are of concern after they have submitted their answers constitutes an unacceptable risk. Researchers often tell us that this is too difficult to do, although others seem able to manage. If, however, it is too difficult to do, I would see the research as presenting an unacceptable risk.

Reference 10 - 1.12% Coverage

Risk needs to be ’kept in place’ - all to often it becomes a substitute for ethics review

Reference 11 - 1.12% Coverage

I felt that the idea of ’risk’ was focused on very overt risks more than subtle ones like social and economic risk.

Reference 12 - 1.12% Coverage

study designs in and off themselves are not risky, they are appropriate depending on the research question. Risk is related to how a study is conducted. For that reason most of my responses were 5, neither high/low risk as a sensitive survey with very personal questions is not high risk if conducted correctly.

Reference 13 - 1.12% Coverage

So much depends on the researcher - I would worry more about an individual in a DGH with little supervision than a large well established non - commercial team. Commercial research is also very diverse from one product companies who can be much more focused on their product than the ethics to very large companies where the same can be said, to other much more apparently ethical approaches. Asking about risk without considering benefit is a bit of an unbalanced equation as a the risk does always have to be considered alongside the benefit.

Reference 14 - 1.12% Coverage

I have been looking at the concept of micromorts because they attempt to put risk in context. I think they are useful. I found this survey needed more context for the meaning of the scores. For example I gave the first-in-man study question a rating of 6 when many REC members might think it deserves 10. I have been the PI in several such studies and believe that risk can be factored out, otherwise I would not do them.

Reference 15 - 0.43% Coverage

Risk is not always direct- studies of any design can be damaged by faulty data collection and analysis and if then used to inform practice or policy,participants as well as the wider public are at risk.

Reference 16 - 1.12% Coverage

Risk issues are often understated or indeed omitted from the PIS and this should be addressed in the training given to researchers.

Reference 17 - 1.12% Coverage

just the point that are we talking about risk to participants or risk to researchers. e.g lone worker policy

Reference 18 - 0.96% Coverage

Some questionnaires can raise v sensitive topics or highlight problems for which there are no solutions or generate new concerns. Phase 1 research into a ‘me too’ drug is likely to be less risky than a new class kid drug etc. Depends where & who is doing the research too , are other options available, what is the burden of the research commitment etc. Some questionnaire studies can pose a greater risk than taking a single additional blood sample for research. Who is determining what is considered a risk that is an individual decision.

Reference 19 - 0.87% Coverage

I would always rate clinical risk highest, but that doesn’t mean that other types of risk aren’t as important. The risk of breaching someone’s confidentiality could be high in certain types of study design, and whilst the impact might be different for the participant, it doesn’t make it any less acceptable than placing someone at clinical risk.

Reference 20 - 0.70% Coverage

I found it almost impossible to separate risk from benefit. I also assumed proper professional oversight of all data was in place.

Reference 21 - 1.12% Coverage

I prefer a generally small risk in research instead of higher risk of exposing people to untested interventions i practice. Well planned research is in general less risky than experiments in practice outside research settings.

Reference 22 - 1.12% Coverage

I consider poor study design likely to cause biased findings to be a significant risk. I consider over complex participant info sheets/consent forms to be a risk.

Reference 23 - 1.12% Coverage

Risk is not just affected by the context and benefits but also dependent on the expertise, experience and care of the researcher. Even very low risk research presents a number of risks to the participant if the researcher does not understand ethics

Reference 24 - 1.12% Coverage

Assessment of risk determines the level of conditions required to be applied to a research project when submitted for ethical review.

Reference 25 - 1.12% Coverage

Risk is relative to benefits. For example, it may be relevant to do genetic analysis on an individual, however they need to be informed that findings may also affect their relatives, especially when related to risks of developing certain diseases like Huntington’s for example, and that may have effects on things like life insurance. A phase 1 first in man is by definition riskier than a later phase of a trial. The risk is however different if this is a first in class or a variant of an exiting drug. Likewise interviews can seem quite risk free, however certain types of interviews can elicit strong emotional responses or reveal risks to the individual or others that the researcher has to have procedures to deal with. Observational studies, that seem very mild and low risk, can become high risk if they involve observation of behaviors in homeless drug users, for example. So although theoretically some designs are less "risky" than others, each has to be evaluated on its own merits.
