# Supplementary material for: Ranking Research Methodology by Risk — a cross-sectional study to determine the opinion of research ethics committee members
Source: Syst Rev. 2023 Sep 1;12:154. doi: 10.1186/s13643-023-02295-1 (PMC10472668; doi:10.1186/s13643-023-02295-1)
Supplement: Supplementary file 2 — Additional file 2. Qualitative data: Anonymity. Broader Methodology Concerns. Clinical Trials. Codebook - EVBRES Qu20. Comment on our Survey. Comment on specific study designs. Comments on Risk and Benefit. Conduct of researchers. Confidentiality. Consent. Context. Data & Privacy. Extra question or options needed. Observational Study. PPIE. Questions not specific enough. Reference to national guidance. Response to Reviewers ver2. Surveys. Thanks, appreciation, offer of help. [file 13643_2023_2295_MOESM2_ESM.zip › Conduct of researchersR1.docx]

Files\\EVBRES Qu20 - § 7 references coded [ 5.58% Coverage]

Reference 1 - 0.66% Coverage

The risks are drastically increased if the data gets into the public domain so it is more about the researchers handling of the data rather than the risks to the participants in the collection or research publication of the data. I filled it in assuming data handling was done appropriately

Reference 2 - 0.43% Coverage

Our committee is seeing increasing numbers of studies where psychological screening tools, for eg anxiety and depression, are being used in anonymous online questionnaires. If they were being used in a face to face setting by a qualified practitioner, there are scores that would indicate that the person concerned should be urged to see further help, even though they do not provide a clinical diagnosis.

Reference 3 - 0.43% Coverage

Risk is not always direct- studies of any design can be damaged by faulty data collection and analysis and if then used to inform practice or policy,participants as well as the wider public are at risk.

Reference 4 - 0.69% Coverage

I see far too much proposed analysis that is seriuously flawed and it would be better if the studies concerned were not allowed to start in the first place unless their analytical element is strongly challenged and corrected beforehand as it is the consequences of a flawed conclusion that presents the greatest risk by far

Reference 5 - 1.12% Coverage

It all depends on the way the research team handle the participants and in the next phase the data.

Reference 6 - 1.12% Coverage

I think it depends on the intent and conduct of the researchers

Reference 7 - 1.12% Coverage

Risk is not just affected by the context and benefits but also dependent on the expertise, experience and care of the researcher. Even very low risk research presents a number of risks to the participant if the researcher does not understand ethics
