# Supplementary material for: Ranking Research Methodology by Risk — a cross-sectional study to determine the opinion of research ethics committee members
Source: Syst Rev. 2023 Sep 1;12:154. doi: 10.1186/s13643-023-02295-1 (PMC10472668; doi:10.1186/s13643-023-02295-1)
Supplement: Supplementary file 2 — Additional file 2. Qualitative data: Anonymity. Broader Methodology Concerns. Clinical Trials. Codebook - EVBRES Qu20. Comment on our Survey. Comment on specific study designs. Comments on Risk and Benefit. Conduct of researchers. Confidentiality. Consent. Context. Data & Privacy. Extra question or options needed. Observational Study. PPIE. Questions not specific enough. Reference to national guidance. Response to Reviewers ver2. Surveys. Thanks, appreciation, offer of help. [file 13643_2023_2295_MOESM2_ESM.zip › ConfidentialityR1.docx]

Files\\EVBRES Qu20 - § 1 reference coded [ 1.12% Coverage]

Reference 1 - 1.12% Coverage

I am not a member of an ethics committee. I am a member of a committee that reviews whether specific uses of confidential patient information without consent would be in the public interest. In my view, weaknesses in study design and in particular uncertainty about the risk that a study may not have enough power to reach a valid conclusion in the public interest, are quite often hard to weigh against the risk of disclosing personal information that could be objectionable to some individuals. I would have more confidence in our recommendations if we could be sure that sponsors took seriously their responsibility not to permit a study to go ahead if it is unlikely to serve any useful purpose.
