# Supplementary material for: Ranking Research Methodology by Risk — a cross-sectional study to determine the opinion of research ethics committee members
Source: Syst Rev. 2023 Sep 1;12:154. doi: 10.1186/s13643-023-02295-1 (PMC10472668; doi:10.1186/s13643-023-02295-1)
Supplement: Supplementary file 2 — Additional file 2. Qualitative data: Anonymity. Broader Methodology Concerns. Clinical Trials. Codebook - EVBRES Qu20. Comment on our Survey. Comment on specific study designs. Comments on Risk and Benefit. Conduct of researchers. Confidentiality. Consent. Context. Data & Privacy. Extra question or options needed. Observational Study. PPIE. Questions not specific enough. Reference to national guidance. Response to Reviewers ver2. Surveys. Thanks, appreciation, offer of help. [file 13643_2023_2295_MOESM2_ESM.zip › ConsentR1.docx]

Files\\EVBRES Qu20 - § 3 references coded [ 2.54% Coverage]

Reference 1 - 1.12% Coverage

Consent becomes highly apposite in the context of full and understandable explanations of potential risk; we are all entitled to choose our risk level provided we are properly informed.

Reference 2 - 0.29% Coverage

I don’t think risk to a participant changes if you gain consent. They might be more comfortable asking questions that may eventually lead to them being at ease with the data collection, but I don’t think this changes the level of risk.

Reference 3 - 1.12% Coverage

In every time of research details of the methods used should be explicitly mentioned and communicated to participants while always giving the option to opt out at any point of time as well have access to analysis and results. In addition to how data is being handled/ transferred/ stored etc
