# Supplementary material for: Ranking Research Methodology by Risk — a cross-sectional study to determine the opinion of research ethics committee members
Source: Syst Rev. 2023 Sep 1;12:154. doi: 10.1186/s13643-023-02295-1 (PMC10472668; doi:10.1186/s13643-023-02295-1)
Supplement: Supplementary file 2 — Additional file 2. Qualitative data: Anonymity. Broader Methodology Concerns. Clinical Trials. Codebook - EVBRES Qu20. Comment on our Survey. Comment on specific study designs. Comments on Risk and Benefit. Conduct of researchers. Confidentiality. Consent. Context. Data & Privacy. Extra question or options needed. Observational Study. PPIE. Questions not specific enough. Reference to national guidance. Response to Reviewers ver2. Surveys. Thanks, appreciation, offer of help. [file 13643_2023_2295_MOESM2_ESM.zip › ContextR1.docx]

Files\\EVBRES Qu20 - § 25 references coded [ 25.43% Coverage]

Reference 1 - 1.12% Coverage

all of the above answers depend on the context, the participants, researchers experience and the potential benefit of the research

Reference 2 - 1.12% Coverage

I think that because of the very broad nature of the questions it was really quite difficult to assess the level of risk. I had a lot of ’what if questions’ in my mind when trying to make the broad assessment.

Reference 3 - 1.12% Coverage

I think risk is hard to contextualise without a proposal to read about mitigating factors. It is also different between different research groups/types of research/locations of research etc. If you are running a covert observational study in public that has different risks associated in comparison to a clinical controlled environment

Reference 4 - 1.12% Coverage

The questions are difficult to address in absence of a context. Some of the answers may have been quite different depending on the circumstances of the research.

Reference 5 - 1.12% Coverage

Context is everything and when reviewing applications I assess all the parameters including documents, methods etc in context before coming to a decision including applicants experiance as well as supervisors experiance

Reference 6 - 1.12% Coverage

For some of the binary questions, I wanted to answer yes and no. Context is important. I think that no human endeavour is entirely free of risk, hence I didn’t use 1.

Reference 7 - 1.12% Coverage

It was difficult to make assessments in the absence of context e.g. in the case of FIH clinical trials. Questions would be what is the agent being studied?

Reference 8 - 0.34% Coverage

Risk varies more by the actual data being collected, than by the context.

Reference 9 - 1.12% Coverage

I would also think risk is very dependent on the target group as well.

Reference 10 - 1.12% Coverage

Understandably difficult to quantify as much depends on context.

Reference 11 - 1.12% Coverage

some of the assessments are based on generalised viewpoints[ in actual situations more detail of risk mitigation would be taken into account, especially the security of the deidentification of data.

Reference 12 - 1.12% Coverage

I feel regardless of the benefits of the study, the risks of research design is impossible to assess on its own - other contextual factors play a role in this determination (e.g the participants cohort/vulnerability, their recruitment, the burden of participation, etc).

Reference 13 - 0.21% Coverage

Well designed and supervised phase 1 clinical trial can be less risky than a questionnaire depending on the circumstances.

Reference 14 - 1.12% Coverage

So much depends on the researcher - I would worry more about an individual in a DGH with little supervision than a large well established non - commercial team. Commercial research is also very diverse from one product companies who can be much more focused on their product than the ethics to very large companies where the same can be said, to other much more apparently ethical approaches. Asking about risk without considering benefit is a bit of an unbalanced equation as a the risk does always have to be considered alongside the benefit.

Reference 15 - 1.12% Coverage

I have been looking at the concept of micromorts because they attempt to put risk in context. I think they are useful. I found this survey needed more context for the meaning of the scores. For example I gave the first-in-man study question a rating of 6 when many REC members might think it deserves 10. I have been the PI in several such studies and believe that risk can be factored out, otherwise I would not do them.

Reference 16 - 1.12% Coverage

Risk is based on the whole study, the details, the location, the experience of the study team etc, so difficult to quantify in a questionnaire

Reference 17 - 1.12% Coverage

In many cases the answers are extremely dependent on other factors, like anonymity, disclosure, confidentiality, and on the nature of the study intervention (not just the study design), eg an RCT of a new vaccine, vs an RCT of a new format for patient information; the level of vulnerability of the study population; etc. It is very difficult to make a judgement or risk devoid of this information.

Reference 18 - 1.12% Coverage

I assess risk when looking at all parameters including presented paperwork so difficult to respond to you’re questions

Reference 19 - 1.12% Coverage

I think you sacrifice too much when trying to quantify risk to research participants in this manner. It could even hinder and misdirect. Sensitivity to the variables involved, such as context and content is incredibly important in developing a useful and grounded understanding. An illustration of reductionism such as this is risky.

Reference 20 - 0.17% Coverage

Risk is v Context dependant so answering these questions is almost like being blindfolded.

Reference 21 - 1.12% Coverage

The research design may well be a context associated with risk to research participants per se.

Reference 22 - 1.12% Coverage

As usual when discussing ethical issues, my response to most of these questions where you force a choice with relatively little information is ’it depends on the specific circumstances’ and so found this quite difficult.

Reference 23 - 1.12% Coverage

I wasn’t always sure how to interpret ’risky’ - but usually took this to mean ’risk of harm’, I think. Yes - many of my answers could have changed quite a bit if more parameters and design factors had been specified, e.g. chance of re-identifiability of anonymised data sets, sensitivity of topic in public observations, etc.

Reference 24 - 1.12% Coverage

Frustrating survey because risk is contextually driven. Observation in public spaces for example could include watching drug deals (higher risk) versus seeing how people interact with a public park (lower risk).

Reference 25 - 1.12% Coverage

I frequently would have liked to answer ’it depends’ when it comes to the level of risk. We can never accurately anticipate the ways in which research may negatively impact individuals, each with unique lived experience and physical, emotional and psychological make-up and associated trigger points.
