# Supplementary material for: Ranking Research Methodology by Risk — a cross-sectional study to determine the opinion of research ethics committee members
Source: Syst Rev. 2023 Sep 1;12:154. doi: 10.1186/s13643-023-02295-1 (PMC10472668; doi:10.1186/s13643-023-02295-1)
Supplement: Supplementary file 2 — Additional file 2. Qualitative data: Anonymity. Broader Methodology Concerns. Clinical Trials. Codebook - EVBRES Qu20. Comment on our Survey. Comment on specific study designs. Comments on Risk and Benefit. Conduct of researchers. Confidentiality. Consent. Context. Data & Privacy. Extra question or options needed. Observational Study. PPIE. Questions not specific enough. Reference to national guidance. Response to Reviewers ver2. Surveys. Thanks, appreciation, offer of help. [file 13643_2023_2295_MOESM2_ESM.zip › Data & PrivacyR1.docx]

Files\\EVBRES Qu20 - § 7 references coded [ 6.74% Coverage]

Reference 1 - 1.12% Coverage

I was unclear of the nature of the risk, I know it was about wellbeing, physical and mental to the participants but areas such as secondary data the risks are low to participnats if hte data is handles well. The risks are drastically increased if the data gets into the public domain so it is more about the researchers handling of the data rather than the risks to the participants in the collection or research publication of the data. I filled it in assuming data handling was done appropriately

Reference 2 - 1.12% Coverage

some of the assessments are based on generalised viewpoints[ in actual situations more detail of risk mitigation would be taken into account, especially the security of the deidentification of data.

Reference 3 - 1.12% Coverage

Technology is outpacing our knowledge of risk. Especially in relation to data linkage. Sometimes people’s privacy needs to be safeguarded and respected even though there is no discernible ‘harm”

Reference 4 - 0.53% Coverage

In many cases the answers are extremely dependent on other factors, like anonymity, disclosure, confidentiality, and on the nature of the study intervention (not just the study design)

Reference 5 - 0.59% Coverage

The risk of breaching someone’s confidentiality could be high in certain types of study design, and whilst the impact might be different for the participant, it doesn’t make it any less acceptable than placing someone at clinical risk

Reference 6 - 1.12% Coverage

There is a relationship in many people eyes between privacy and risk which I have factored into my answers

Reference 7 - 1.12% Coverage

You didn’t mention if the audio and video interviews would be recorded and/or transcribed later. I see higher risk there as participant’s data could be accessed by others any other time and info could be stolen.
