# Supplementary material for: Ranking Research Methodology by Risk — a cross-sectional study to determine the opinion of research ethics committee members
Source: Syst Rev. 2023 Sep 1;12:154. doi: 10.1186/s13643-023-02295-1 (PMC10472668; doi:10.1186/s13643-023-02295-1)
Supplement: Supplementary file 2 — Additional file 2. Qualitative data: Anonymity. Broader Methodology Concerns. Clinical Trials. Codebook - EVBRES Qu20. Comment on our Survey. Comment on specific study designs. Comments on Risk and Benefit. Conduct of researchers. Confidentiality. Consent. Context. Data & Privacy. Extra question or options needed. Observational Study. PPIE. Questions not specific enough. Reference to national guidance. Response to Reviewers ver2. Surveys. Thanks, appreciation, offer of help. [file 13643_2023_2295_MOESM2_ESM.zip › Extra question or options neededR1.docx]

Files\\EVBRES Qu20 - § 8 references coded [ 7.62% Coverage]

Reference 1 - 1.12% Coverage

Slight flaw in that clinical research is not the only type of academic research. Maybe, there should have been an extra question: "Is your research experience entirely clinical, partly clinical or not at all clinical?"

Reference 2 - 0.46% Coverage

I’m a retired clinician - nowhere to say this in the survey

Reference 3 - 1.12% Coverage

The survey should study research where non consenting bystanders may be unwittingly involved and privacy compromised and research that involves facial recognition.

Reference 4 - 1.12% Coverage

The survey does not allow for a retired option

Reference 5 - 1.12% Coverage

Please note I am retired from paid employment but that doesn’t seem to be an option in the early questions. I indicated that I don’t conduct research but I have done so, until quite recently. These days, clinician includes allied health professionals such as nurses, pharmacists, dentists, physios etc. Was that your meaning?

Reference 6 - 0.42% Coverage

This survey assumes someone is still employed. Many REC members are retired.

Reference 7 - 1.12% Coverage

In the question about where is your employer based, followed by a list of countries, I am not employed so I did not answer that. An additional option may be useful in future studies.

Reference 8 - 1.12% Coverage

You have not included in this questionnaire the risk perception of studies involving stem cells. I consider research involving the extraction of pluripotent and research administering artifically grown cells from stem cells currently to be at the highest risk level, thus a 10.
