# Supplementary material for: Ranking Research Methodology by Risk — a cross-sectional study to determine the opinion of research ethics committee members
Source: Syst Rev. 2023 Sep 1;12:154. doi: 10.1186/s13643-023-02295-1 (PMC10472668; doi:10.1186/s13643-023-02295-1)
Supplement: Supplementary file 2 — Additional file 2. Qualitative data: Anonymity. Broader Methodology Concerns. Clinical Trials. Codebook - EVBRES Qu20. Comment on our Survey. Comment on specific study designs. Comments on Risk and Benefit. Conduct of researchers. Confidentiality. Consent. Context. Data & Privacy. Extra question or options needed. Observational Study. PPIE. Questions not specific enough. Reference to national guidance. Response to Reviewers ver2. Surveys. Thanks, appreciation, offer of help. [file 13643_2023_2295_MOESM2_ESM.zip › Observational StudyR1.docx]

Files\\EVBRES Qu20 - § 2 references coded [ 1.61% Coverage]

Reference 1 - 1.12% Coverage

Observational studies in public spaces can put research at risk if approached by a member of the public or police if researcher’s behaviour appears unusual and they may need to mitigate for this and have information about the study they can provide to reassure

Reference 2 - 0.48% Coverage

If you are running a covert observational study in public that has different risks associated in comparison to a clinical controlled environment
