# Supplementary material for: Ranking Research Methodology by Risk — a cross-sectional study to determine the opinion of research ethics committee members
Source: Syst Rev. 2023 Sep 1;12:154. doi: 10.1186/s13643-023-02295-1 (PMC10472668; doi:10.1186/s13643-023-02295-1)
Supplement: Supplementary file 2 — Additional file 2. Qualitative data: Anonymity. Broader Methodology Concerns. Clinical Trials. Codebook - EVBRES Qu20. Comment on our Survey. Comment on specific study designs. Comments on Risk and Benefit. Conduct of researchers. Confidentiality. Consent. Context. Data & Privacy. Extra question or options needed. Observational Study. PPIE. Questions not specific enough. Reference to national guidance. Response to Reviewers ver2. Surveys. Thanks, appreciation, offer of help. [file 13643_2023_2295_MOESM2_ESM.zip › PPIER1.docx]

Files\\EVBRES Qu20 - § 2 references coded [ 1.78% Coverage]

Reference 1 - 0.65% Coverage

Did you involve research participants (or other PPI) in the design of the survey?

Reference 2 - 1.12% Coverage

I was hoping that participatory designs might have been included in the study as this affected how I answered the earlier question about risk in all kinds of design. When studies can include participants in their design then this empowerment/opportunity for voice can reduce the risk.
