# Supplementary material for: Ranking Research Methodology by Risk — a cross-sectional study to determine the opinion of research ethics committee members
Source: Syst Rev. 2023 Sep 1;12:154. doi: 10.1186/s13643-023-02295-1 (PMC10472668; doi:10.1186/s13643-023-02295-1)
Supplement: Supplementary file 2 — Additional file 2. Qualitative data: Anonymity. Broader Methodology Concerns. Clinical Trials. Codebook - EVBRES Qu20. Comment on our Survey. Comment on specific study designs. Comments on Risk and Benefit. Conduct of researchers. Confidentiality. Consent. Context. Data & Privacy. Extra question or options needed. Observational Study. PPIE. Questions not specific enough. Reference to national guidance. Response to Reviewers ver2. Surveys. Thanks, appreciation, offer of help. [file 13643_2023_2295_MOESM2_ESM.zip › Questions not specific enoughR1.docx]

Files\\EVBRES Qu20 - § 10 references coded [ 10.32% Coverage]

Reference 1 - 1.12% Coverage

Some of these questions were difficult to answer because the examples given were so broad that it was effectively impossible to ascertain risk, e.g. F2F FG: the risk here depends very much on the participants themselves (i.e. their mental state, their inter-relationships, etc.) and the questions being asked. This necessitated guesstimating, rather than estimating - which is unlikely to lead to meaningful findings...

Reference 2 - 1.12% Coverage

I missed the opportunity to say ’sometimes’ rather than yes or no on some of the questions. A number of times my response would have been ’it depends’ - as it is I think the questionnaire will give only a broad brush and rather simplistic analysis of risk appreciation related to research participants

Reference 3 - 1.12% Coverage

Q4 - data only research - what does this mean? Q7 and Q12 - risk to whom, participant, researcher or both? Q10 , 1 - what therapies? the risk would vary so widely that the answer to this question without clarification is meaningless

Reference 4 - 1.12% Coverage

Some questions were a bit hard to answer - e.g. there would seem to be little point in collecting data without being able to identify participants, as it would severely limit the comparisons that could be made and the conclusions drawn.

Reference 5 - 1.12% Coverage

Unable to make any nuanced response with categorical choices

Reference 6 - 0.21% Coverage

Your questionnaire does not provide the option to respond "I don’t know" or "not applicable" and thus creates a serious biais.

Reference 7 - 1.12% Coverage

As usual when discussing ethical issues, my response to most of these questions where you force a choice with relatively little information is ’it depends on the specific circumstances’ and so found this quite difficult.

Reference 8 - 1.12% Coverage

I wasn’t always sure how to interpret ’risky’ - but usually took this to mean ’risk of harm’, I think. Yes - many of my answers could have changed quite a bit if more parameters and design factors had been specified, e.g. chance of re-identifiability of anonymised data sets, sensitivity of topic in public observations, etc.

Reference 9 - 1.12% Coverage

Frustrating survey because risk is contextually driven. Observation in public spaces for example could include watching drug deals (higher risk) versus seeing how people interact with a public park (lower risk).

Reference 10 - 1.12% Coverage

You didn’t mention if the audio and video interviews would be recorded and/or transcribed later. I see higher risk there as participant’s data could be accessed by others any other time and info could be stolen.
