# Supplementary material for: Ranking Research Methodology by Risk — a cross-sectional study to determine the opinion of research ethics committee members
Source: Syst Rev. 2023 Sep 1;12:154. doi: 10.1186/s13643-023-02295-1 (PMC10472668; doi:10.1186/s13643-023-02295-1)
Supplement: Supplementary file 2 — Additional file 2. Qualitative data: Anonymity. Broader Methodology Concerns. Clinical Trials. Codebook - EVBRES Qu20. Comment on our Survey. Comment on specific study designs. Comments on Risk and Benefit. Conduct of researchers. Confidentiality. Consent. Context. Data & Privacy. Extra question or options needed. Observational Study. PPIE. Questions not specific enough. Reference to national guidance. Response to Reviewers ver2. Surveys. Thanks, appreciation, offer of help. [file 13643_2023_2295_MOESM2_ESM.zip › Reference to national guidanceR1.docx]

Files\\EVBRES Qu20 - § 1 reference coded [ 1.12% Coverage]

Reference 1 - 1.12% Coverage

Determination of risk in part is guided by the Australian National Health and Medical Research standards. However, risk must be determined on more than methodology. Inappropriate data management plans and publication can make low risk methodological studies into ethical nightmares. Applications must be reviewed on a case by case basis. The HERA form does have some initial checks for researchers to decide risk level prior submission. These studies are reviewed in our case by two members out of session. If the researchers have misidentified their level of risk the study goes to a whole of committee meeting.
