# Supplementary material for: Ranking Research Methodology by Risk — a cross-sectional study to determine the opinion of research ethics committee members
Source: Syst Rev. 2023 Sep 1;12:154. doi: 10.1186/s13643-023-02295-1 (PMC10472668; doi:10.1186/s13643-023-02295-1)
Supplement: Supplementary file 2 — Additional file 2. Qualitative data: Anonymity. Broader Methodology Concerns. Clinical Trials. Codebook - EVBRES Qu20. Comment on our Survey. Comment on specific study designs. Comments on Risk and Benefit. Conduct of researchers. Confidentiality. Consent. Context. Data & Privacy. Extra question or options needed. Observational Study. PPIE. Questions not specific enough. Reference to national guidance. Response to Reviewers ver2. Surveys. Thanks, appreciation, offer of help. [file 13643_2023_2295_MOESM2_ESM.zip › Response to Reviewers ver2R1.docx]

School of Health and Care Professions

University of Portsmouth

Portsmouth, UK

29^th^ June 2023

Dear Dr Balloso,

We would like to thank you and reviewer 2 for their very positive comments on our manuscript “*Ranking Research Methodology by Risk – the Opinion of Research Ethics Committees”* (SYSR-D-23-00199). However, we did struggle a bit in addressing the comments of Reviewer 1, as they are extensive, and in many places contradictory to reviewer 2.

In trying to balance this contradiction we have made significant edits to the manuscript, trying to address the edits requested by reviewer 1 as much as possible. As for our discussion section, we need to clarify that the major purpose of this project described here, as funded by the European Commission and conducted by the EVBRES (Evidence Based Research) team, was to produce ***guidance*** specifically for RECs as to the types of literature reviews they might expect for different types of research. In considering how to create such guidance, and what empirical evidence could be used or gathered to inform this, we settled on proposing a risk hierarchy linked to methodology as we now describe. The idea is that this evidence can be used to infer, or guide, RECs consideration of evidence being used to justify the project(s) they are asked to review. We have not drawn any stronger a link than this, have been entirely open about not being definitive, and discuss this at length. We are happy to see that reviewer 2 praised us for this openness. The complex nature of REC review means that “concrete” instructions are seldom appropriate, and instead RECs need guidance to help them reflect on the best decision to make given the study under review. Indeed, during our extensive discussions conducted over a number of years as part of the EVBRES project, we concluded that it is very difficult to conceive of a study that directly links specific review type to specific methodology as there are simply too many relevant parameters. As such we settled on a study that focused specifically on the parameter of risk relating to methodology which, as we demonstrate, can be addressed empirically, before making subsequent inferences as to how this can be linked to different review types, that we discuss at length in the discussion. It is now up to members of RECs to read our paper, and use this guidance as they see fit!

Except for this clarification regarding the outcome of our project, and how we have described this in our discussion, we have followed the majority of the rest of reviewer 1’s suggestions, and thank the reviewer for their time invested in the improvement of our manuscript. Please also find below an explanation of how we have addressed each comment (reviewers comments in bold, italic):

***Reviewer #1:***

***Title***

***-In the title, it is unclear what kind of risk the authors are referring to; I suggest to revise to properly describe the type of risk.***

***-The title is missing a study design descriptor. It should be added at the end of the title that this was a cross-sectional study.***

***-"hierarchy of risk" sounds like a good term to mention in the title***

We thank the reviewer for the suggestion and have added that this was a cross-sectional study. We felt that an explanation of the type of risk, or the fact that a hierarchy had been created, would make the study title too long.

***Abstract***

***-Study design should be described at the beginning of the methods.***

***-Time when the study was conducted should be specified.***

***-Eligible participants should be described.***

***-Abstract is rather brief. Systematic Reviews journal allows up to 350 words in the abstract (quote: "The Abstract should not exceed 350 words."). Thus, the authors are advised to use all this space.***

***-The aim at the end of the Introduction says "and then gained qualitative data" - but this is not mentioned in the abstract.***

We thank the reviewer for their comments and have added the requested information and extended the abstract.

***Introduction***

***-"A formal Systematic Review…" - no need to write this with capital letters.***

***-"This is because there is simply no time or funding to produce…" - but maybe it already exists.***

***-"While RECs expecting to see…" - please provide a reference to the study indicating that this is what they expect. I am member of a REC and I have yet to see that my colleagues in the REC expect this.***

***-the EVBRES - this is casually mentioned in the abstract, but without any details about the project. I suggest to devote one paragraph to the COST EVBRES and to provide a reference to the action website.***

***-"The aim of this study was therefore to explore this idea empirically…" - instead of referring to "this idea", please write the aim in full.***

***-"and then gained qualitative data" - this is not mentioned in the abstract.***

We have updated the introductory section as suggested above.

***Methods***

***-Please add the first subtitle/subsection titled Study design, and provide information that this was a cross-sectional study.***

***-Please add another subtitle "Protocol" to describe that the protocol was developed a priori and please provide information about whether it was published anywhere.***

***-Please add the third subtitle "Ethics" and bring forward information about the ethics here, to be a standalone and prominent information.***

We have created a sub heading at the beginning of the methods to state the study design, and also added a sentence about the protocol being developed *a priori*. The ethics section is clearly labelled further down the methods section, which as written systematically describes the method we used to develop the research. As such the ethics approval was obtained after the survey/questionnaire was designed, and thus we feel that the section makes more sense to remain where it is.

***-Questionnaire design: write explicitly that this was a newly designed questionnaire. Also, please indicate immediately that the full questionnaire is available in the supplementary file. And then, please, also provide the questionnaire in full as a standalone supplementary file (currently, only a part of the questionnaire is provided in the supplementary file). Different types of information should be provided as separate supplementary files (and numbered, and referred to in the text).***

We have added that the full questionnaire can be found in the supplemental information, and that the questionnaire was designed specifically for this study.

***-"Discussions…" - Please cite also literature that was used together with discussions. I assume you did not start only from discussions?***

We have added a reference to a Cochrane article on the topic.

***-Page 15, "We acknowledge" - it is unclear why is this in italics and quotation marks.***

***-Table 2: please explain all acronyms below the table***

We have clarified that the text in quotation were definitions added into the questionnaire to help respondents. Regarding table 2, all acronyms are expanded on first use.

***-Please provide more methodological details about the qualitative data analysis.***

***-The last section of methods should include a subtitle called Raw data.***

We have now included the qualitative data in the supplementary materials.

***Results***

***Figures 1, 2 and 3 should be merged into a single table. There is no advantage of these figures over a table. Particularly it is confusing that not all the "columns" are proportional relative to the 100% so they may give different perception of results to the readers. For example, column showing 80% is equal to the column showing 44% and 34%. This is not OK.***

This has been done as table 3 for the characteristics of survey respondents, although we have kept the figure relating to anonymity as here the columns are equivalent.

***Qualitative data are described only with 2 sentences, none of which is presenting results. This part needs to be presented in more detail in text, with quotations. Often it is unclear from the Table what the participants were referring to.***

We have added a column to the table providing an illustrative statement for each category.

***Discussion***

***"They should seek pragmatic alternatives…" - the authors give advice to the RECs that is not in line with the principles of EBR. The authors indicate that different evidence syntheses could be adequate for different perceived risks, but they do not support their advice with literature references and this advice is very concerning. The authors did not conduct a study about which type of evidence synthesis would be acceptable for different risk hierarchies. Thus, the authors should rewrite their entire discussion to avoid giving such advice. The same comment goes for the parts of Figure 4 and 5. The authors should remove their recommendations about which evidence should be considered adequate to justify new research, based on risk hierarchies. This is opinion of authors, that this is sufficient, not a consensus in the research community.***

Here there is a contradiction of the opinion of reviewer 1 and reviewer 2. Indeed we carefully caveat our discussion to demonstrate that the research described here only provides empirical data to create the hierarchy of risk, and our comments on how this carries over to the type of review that might be required is clearly stated as “guidance” and “an indication”. Indeed to quote reviewer 2 verbatim: “*The authors acknowledge their overall approach as very general and broad. I agree with that assessment, but I also agree that although the approach is very general, it is a very good starting point in looking at proposed research in a way that does not necessitate a high degree of prior knowledge about the proposed research. It does not take to the onus away from the researcher to have a good reason to propose certain research, but it lightens the burden of the reviewers to make more of an informed decision about proposed research*.”

***Conclusion***

***-Word "concrete" would better be replaced with "specific"***

Here we agree with the referee that language choice is very important. Therefore as the overall aim of this part of the EVBRES project was to produce guidance *specific* to risk and methodology, we feel that the guidance is indeed *specific* but not, as we say in the text, “concrete” in the sense of being firm and immovable. We would therefore be keen to keep this word if the reviewer agrees.

***-"While it would be a mistake for RECs to demand the type of review we suggest" - it is unclear why this would be a mistake? Principles of evidence-based research suggest that a systematic review should be sought.***

We think that there is some contradiction in this remark with earlier reviewer’s comments in relation to the results.

***-"in an appropriately systematic way" - many things that the authors are advising for different risk hierarchy levels are not "appropriately systematic" so the authors are being contradictory.***

This statement refers to the RECs making a decision that is “appropriately systematic”.

***Availability of data and materials***

***The authors wrote "The datasets used and/or analysed during the current study are available from the corresponding author on reasonable request. "***

***Could you please provide your raw data as a supplementary file, or publish the raw data in a publicly available repository (and then share the link in the manuscript)? The data you generated do not appear sensitive in a way that could not be shared….***

This has been done.

***Funding***

***Who will pay for the APC for this manuscript? That source of funding should be acknowledged here.***

This is now stated.

***References***

***The references are very messy and inconsistent.***

The references have been entered using the Mendeley database and the built-in “Vancouver” template that we believe reflects the journal’s style. We would, of course, follow the editor’s guidance should a different format be required. However, we do agree that with almost all journals now being published online, there are certain inconsistencies as to how articles are now displayed, meaning that page numbers are simply not used in many journals. Similarly a DOI link is increasingly the most reliable way to reference a wide range of sources. We have therefore looked to the Mendeley software to adjudicate this variability.

***Reviewer #2: …I have enjoyed reading the article and think it is a very good starting point for further research in this particular area of patient safety, risk, risk evaluation and specific role Ethics Committees and similar bodies have in their role of safeguarding patients and researchers alike. I especially commend the one page practical guide at the end because it highlights the findings of the authors very well and provides a great and easy tool for REC members to use, regardless of their own area of expertise… The proposed question is highly relevant to ensure patients safety and a purposeful use of resources…***.

We thank reviewer 2 for their comments (which continue along the same vein), and just would wish to point out that we tried our best to balance both reviewers’ opinions, especially in relation to the discussion and our one-page guidance sheet for RECs.

We hope that the extensive changes we have made to the manuscript have now improved the paper, which we are keen to publish as a major outcome of our four year EVBRES project.

Thank you for your time reviewing our manuscript and response,

Simon Kolstoe

Jennifer Durning

Jennifer Yost

Silviya Aleksandrova -Yankulovska
