# Supplementary material for: Ranking Research Methodology by Risk — a cross-sectional study to determine the opinion of research ethics committee members
Source: Syst Rev. 2023 Sep 1;12:154. doi: 10.1186/s13643-023-02295-1 (PMC10472668; doi:10.1186/s13643-023-02295-1)
Supplement: Supplementary file 2 — Additional file 2. Qualitative data: Anonymity. Broader Methodology Concerns. Clinical Trials. Codebook - EVBRES Qu20. Comment on our Survey. Comment on specific study designs. Comments on Risk and Benefit. Conduct of researchers. Confidentiality. Consent. Context. Data & Privacy. Extra question or options needed. Observational Study. PPIE. Questions not specific enough. Reference to national guidance. Response to Reviewers ver2. Surveys. Thanks, appreciation, offer of help. [file 13643_2023_2295_MOESM2_ESM.zip › SurveysR1.docx]

Files\\EVBRES Qu20 - § 1 reference coded [ 1.12% Coverage]

Reference 1 - 1.12% Coverage

Our committee is seeing increasing numbers of studies where psychological screening tools, for eg anxiety and depression, are being used in anonymous online questionnaires. If they were being used in a face to face setting by a qualified practitioner, there are scores that would indicate that the person concerned should be urged to see further help, even though they do not provide a clinical diagnosis. I (as does the committee on which I serve) see asking people to reflect on their psychological wellbeing increases the risk that someone might self-harm. It is also likely that people who are concerned about their psychological well-being are more likely to answer this kind of survey, so not providing participants with an indication of what kind of responses are of concern after they have submitted their answers constitutes an unacceptable risk. Researchers often tell us that this is too difficult to do, although others seem able to manage. If, however, it is too difficult to do, I would see the research as presenting an unacceptable risk.
