# Supplementary material for: Ranking Research Methodology by Risk — a cross-sectional study to determine the opinion of research ethics committee members
Source: Syst Rev. 2023 Sep 1;12:154. doi: 10.1186/s13643-023-02295-1 (PMC10472668; doi:10.1186/s13643-023-02295-1)
Supplement: Supplementary file 2 — Additional file 2. Qualitative data: Anonymity. Broader Methodology Concerns. Clinical Trials. Codebook - EVBRES Qu20. Comment on our Survey. Comment on specific study designs. Comments on Risk and Benefit. Conduct of researchers. Confidentiality. Consent. Context. Data & Privacy. Extra question or options needed. Observational Study. PPIE. Questions not specific enough. Reference to national guidance. Response to Reviewers ver2. Surveys. Thanks, appreciation, offer of help. [file 13643_2023_2295_MOESM2_ESM.zip › Thanks, appreciation, offer of helpR1.docx]

Files\\EVBRES Qu20 - § 9 references coded [ 7.95% Coverage]

Reference 1 - 1.12% Coverage

I remember a paper linking perceptions of risk of research related to common everyday events. Do you have this? I could find the link if you want

Reference 2 - 1.12% Coverage

Thank you

Reference 3 - 1.12% Coverage

Glad you are examining these matters

Reference 4 - 0.26% Coverage

I would be interested in the report, and would be particularly interested in the models of risk you are using (eg Andy Stirling?). Because within responses there will certainly be ambiguity - did a respondent interpret the question as being bland observation in a public place or looking at drug deals going down.

Reference 5 - 1.12% Coverage

Well presented and structured survey

Reference 6 - 0.43% Coverage

The study falls under the growing trend of "empirical studies" in bioethics and one can wonder the nature of the conclusions you could draw from it. I would be happy to discuss this issue if you wish. Best regards, Dominique Sprumont Dominique.Sprumont@vd.ch

Reference 7 - 1.12% Coverage

Easy to use on phone. Click and turn the page

Reference 8 - 1.12% Coverage

Thank you for this research. I look forward to reading the published articles upon completion.

Reference 9 - 0.52% Coverage

But a really useful survey - look forward to findings. The questions are highly relevant to our research ethics decision-making.
